# Do CBCT and Clinical Experience Impact Decision‐Making in Endodontic Diagnosis and Treatment Planning? A Before‐After Study

**DOI:** 10.1111/aej.12966

**Published:** 2025-06-19

**Authors:** Tamyres Veleda Fonseca, Gabriel Lima Braz, Henrique Timm Vieira, Nadia de Souza Ferreira, Melissa Feres Damian

**Affiliations:** ^1^ Federal University of Rio Grande do Sul Porto Alegre Rio Grande do Sul Brazil; ^2^ Federal University of Pelotas Pelotas Rio Grande do Sul Brazil; ^3^ Department of Semiology and Clinics Federal University of Pelotas Pelotas Rio Grande do Sul Brazil

**Keywords:** clinical decision‐making, cone‐beam computed tomography, dental student, radiography dental, root canal therapy

## Abstract

This before‐after study evaluated the impact of cone beam computed tomography (CBCT) and clinical experience on diagnosis and treatment planning for endodontic cases, compared to periapical radiography (PR). 28 CBCT cases with corresponding PR images were assessed by five endodontic specialists and five final‐year dental students in two stages, 30 days apart: first using PR images and then CBCT scans. McNemar and Wilcoxon signed‐rank tests were applied, along with binary and ordinal logistic regressions, to assess changes in diagnosis and therapeutic decisions between imaging modality, evaluator experience, and perceived difficulty. Most evaluators changed their diagnostic and treatment decisions from PR to CBCT, particularly among students. Evaluator experience significantly influenced the difficulty of diagnosis and treatment planning, especially with CBCT. The interpretation of CBCT images increased decision‐making difficulty for students while facilitating it for professionals. CBCT significantly influenced clinical decisions and perceived difficulty, with effects varying based on evaluator experience.

## 1 Introduction

In Endodontics, as in other areas of Dentistry, imaging examinations are part of a comprehensive system whose ultimate goal is to provide effective patient care. To be considered of high quality, these examinations must demonstrate not only diagnostic efficacy but also therapeutic and even social efficacy [1, 2]. Periapical radiographs (PR) stand as the primary imaging method in Endodontics due to their good resolution, minimal distortion, affordability, and low radiation dose [3, 4]. However, their two‐dimensional nature often results in overlapping structures, complicating their interpretation [5].

Cone beam computed tomography (CBCT) has transformed endodontic imaging by providing high‐resolution, three‐dimensional examination that eliminates structural overlap, allowing for a more accurate assessment of the complex root canal system, as well as the detection of pathological or anatomical alterations [6]. However, its higher X‐ray exposure and susceptibility to artefacts from dense materials, such as root canal filling materials, must be considered [6, 7]. For this reason, the European Society of Endodontology (ESE), the American Association of Endodontists (AAE), and the American Academy of Oral and Maxillofacial Radiology (AAOMR) recommend CBCT only when conventional radiography is inconclusive, particularly in cases involving complex root anatomy, surgical planning, or suspected root resorptions and fractures [3, 8].

Effective clinical decision‐making relies on accurate and practical diagnostic tools [9]. This process is often associated with the clinician's training, experience, and familiarity with imaging methods [10]. Despite the increasing use of CBCT in Endodontics and its robust diagnostic accuracy efficacy, there is still a lack of consensus on how this exam impacts diagnostic thinking and therapeutic efficacy across different levels of clinical experience. Thus, this study aims to address this gap by assessing the impact of CBCT, compared to PR, on the diagnostic and treatment decision‐making of endodontic specialists and final‐year dental students, as well as their perceived difficulty in utilising each imaging modality. Through a variety of scenarios commonly encountered in daily endodontic practice, the findings provide insight into how training and experience shape the interpretation and application of advanced imaging techniques in the context of Endodontics.

## 2 Materials and Methods

### 2.1 Study Design

This single‐center before‐after study was reported in accordance with the adapted version of the QUADAS checklist, for quality assessment of before‐after studies [11]. The research was approved by an institutional Research Ethics Committee and adhered to the ethical principles for biomedical research involving human subjects [12]. Informed consent was obtained from all evaluators involved in the assessments prior to participation. Regarding the use of patient imaging data, the requirement for informed consent was formally waived by the Ethics Committee, considering the retrospective nature of this phase of the study and the complete anonymization of the imaging examinations.

### 2.2 Setting and Participants

Inclusion criteria for the imaging exams included: (1) requiring endodontic intervention/diagnosis and (2) the availability of both CBCT and PR exams. Exclusion criteria included: (1) referrals for non‐endodontic purposes and (2) cases with incomplete root development. Personal identifiers were removed from all scans. To better simulate clinical reality, the selected cases encompassed a variety of scenarios commonly encountered in daily endodontic practice. This approach aimed to reflect the typical heterogeneity of real‐world cases requiring endodontic assessment and intervention.

Based on sample sizes reported in previous studies with similar objectives and methodologies [2, 5, 7, 13], it was aimed to include approximately 30 cases. To reach this number, all CBCT referrals for endodontic purposes submitted to a private radiology clinic (from the clinic's establishment until the end of 2024) were screened by analysing referral information from CBCT requisition forms. Cases were consecutively included as long as they met predefined eligibility criteria. This process continued until the planned sample size was reached. No randomisation, additional filtering, or subjective case selection was applied. This approach corresponds to a non‐probabilistic, convenience sampling strategy, commonly used in studies with retrospective aspects. While this method may limit generalisability, it ensured standardised case selection and supported the internal validity of group comparisons.

The 30 CBCT scans were initially identified by the primary researcher (T.V.F.) based on referral reasons indicating endodontic purposes. Following this, two experienced oral radiologists (M.F.D. and H.T.V.) evaluated the eligibility of the volumes, two cases were excluded for not meeting the inclusion criteria, resulting in a final sample of 28 cases. Next, the specific images to be used in the study were selected. Standardised, high‐quality views were chosen to consistently represent the region of interest, aiming to highlight relevant diagnostic features while minimising redundancy. All selections were subsequently reviewed by an experienced endodontist (N.S.F.) to confirm clinical relevance. None of these professionals were involved in the assessment process.

All CBCT scans included in this study were requested by referring dentists based on justified clinical needs related to endodontic evaluation; specific reasons for referral are available in Table S1. The CBCT exams were performed using the same tomograph PaX‐i3D (Vatech Co, Hwaseong, Gyeonggi, South Korea) with a reduced FOV (5 × 5 cm) and a voxel size of 0.08 mm; accordingly, all PR were acquired using the phosphor plate system VistaScan (Dürr Dental, Bietigheim‐Bissingen, Germany).

**FIGURE 1:**
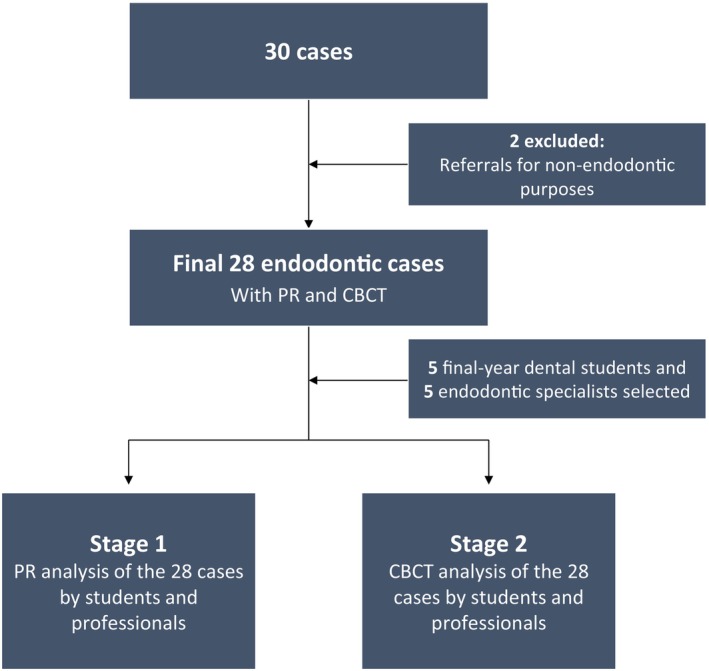
Flowchart of the study's selection and analysis process.

Five final‐year dental students from a public university in southern Brazil and five experienced endodontists (with qualifications in either *Lato* or *Stricto Sensu*) were invited to participate as evaluators in the study. The participating endodontists had a minimum of 5 years of clinical experience and routinely interpret CBCT scans in their professional practice. The undergraduate students underwent curricular training in CBCT interpretation, which included theoretical instruction and supervised pre‐clinical exercises, to ensure adequate familiarity and minimum proficiency prior to their participation in the study. A flowchart of the study's selection and analysis process is available in Figure 1.

### 2.3 Data Sources/Evaluation Process

The evaluation process was conducted in two stages, separated by a one month (30 days) interval in order to minimise the potential influence of maturation bias on decision‐making [5, 14]. In the first stage, evaluators analysed PRs, and in the second, they reviewed CBCT images. The 28 cases were randomly presented in each stage using a Microsoft PowerPoint (.pptx) file (Microsoft Corporation, Redmond, WA, USA), with the cases order shuffled between stages. To eliminate variations caused by different devices, all evaluations were performed on the same HP Celeron 240 G6 notebook (Hewlett‐Packard, Palo Alto, CA, USA) with a 14‐in. screen, individually, in a room with no direct light sources on the monitors and minimal glare, following brief written instructions provided by one of the researchers (T.V.F.).

**FIGURE 2:**
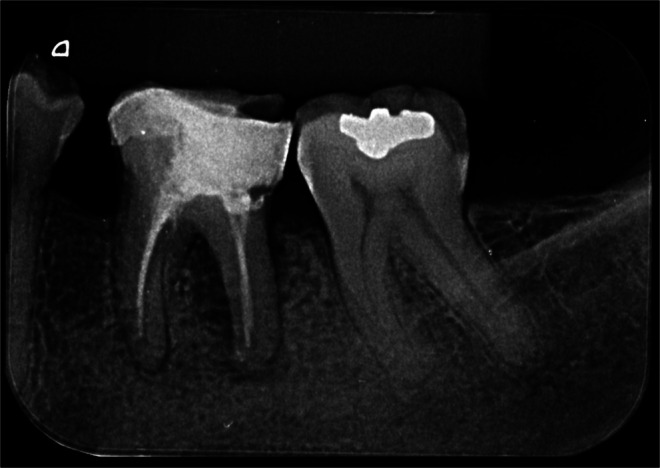
Periapical radiograph (PR) of a left mandibular first molar with previous root canal treatment, showing periradicular radiolucency—interpretation stage 1.

**FIGURE 3:**
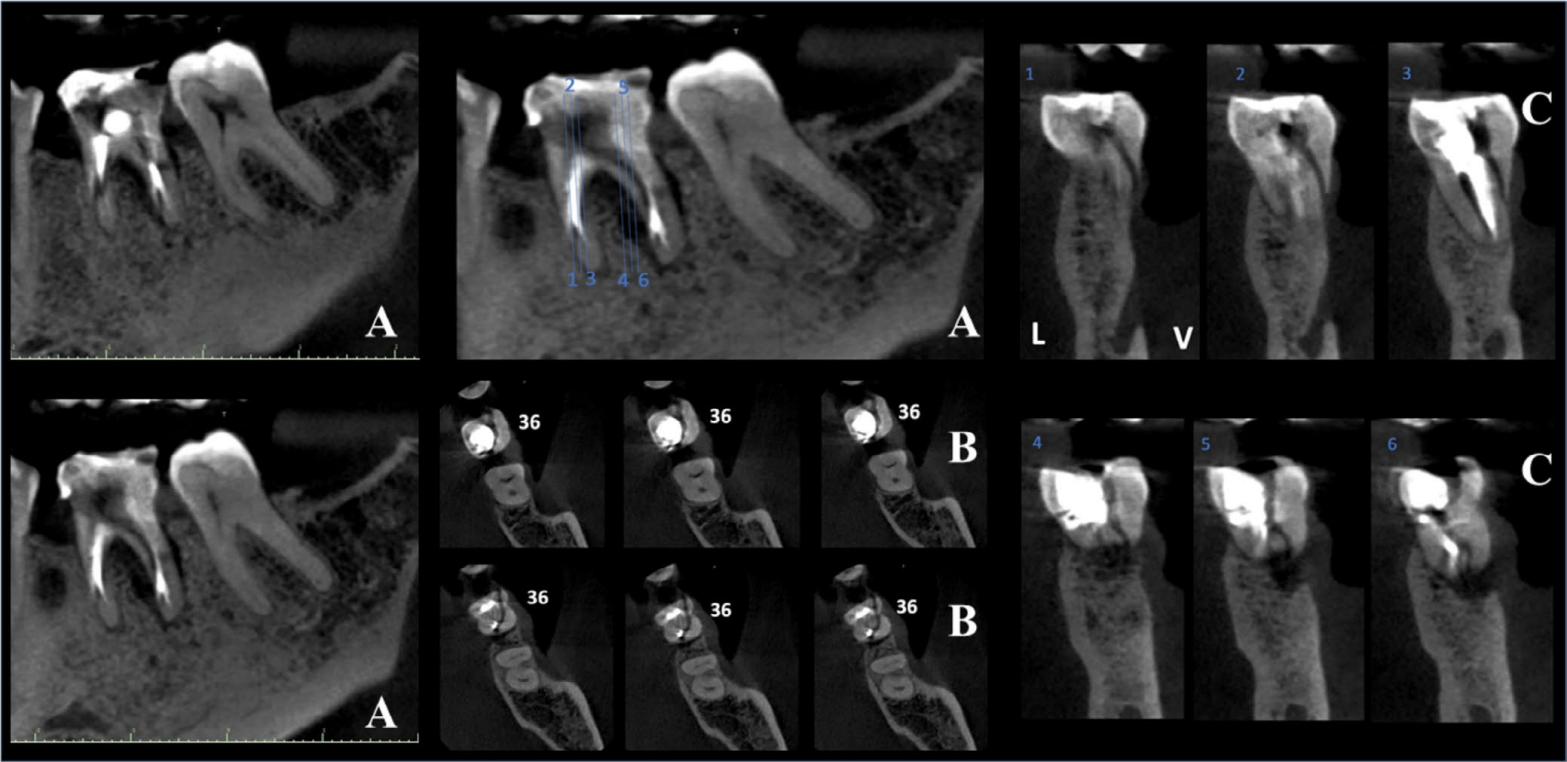
Cone beam computed tomography (CBCT) scan of the same case shown in Figure 2—interpretation stage 2. In addition to the periradicular radiolucency seen in the periapical radiograph, a vertical root fracture is visible in the left mandibular first molar, highlighting the superior detail provided by CBCT imaging in comparison to conventional radiography. Views: Panoramic reconstruction (A), axial slice (B), and oblique slice (C).

In the second stage, cross‐sectional and axial CBCT slices relevant to the evaluated tooth were selected, accompanied by at least one panoramic reconstruction per case. When necessary, additional sagittal and coronal slices were provided. The complete tomographic volume was not made available to ensure that all examiners assessed the same standardised images [15, 16]. To further minimise bias and standardise the interpretation process, evaluators received educational material on CBCT image analysis principles. Examples of the presented cases are illustrated in Figures 2 and 3.

### 2.4 Variables

For each case, the tooth under evaluation was identified, and evaluators were asked to provide both a diagnosis and a proposed treatment plan. Although all cases presented some type of alteration, this detail was intentionally withheld from the evaluators to avoid bias. Their responses were recorded on a standardised form specifically designed for this study. Diagnostic options included: No alteration or pathology (0), Periapical radiolucency (1), Root resorption (2), Root perforation (3), and Root fracture (4). Treatment plan choices were: No treatment needed (0), Conventional root canal treatment (1), Root canal retreatment (2), Perforation repair with root canal treatment (3), Apical surgery (4), and Extraction (5).

If the evaluator deemed it possible to assign more than one diagnosis, the diagnosis should be based on the most significant characteristic of the image, i.e., the one the evaluator considered most apparent among the five options presented. Similarly, if the evaluator believed that more than one treatment option was possible or necessary, the chosen option should be the one with the highest potential to address the case. The changes in diagnostic and treatment planning options after CBCT analysis (second evaluation), compared to periapical radiography analysis (first evaluation), were recorded within each evaluator's clinical experience level group.

Additionally, each evaluator was required to indicate the level of difficulty in assigning the diagnosis and treatment plan for each case. A 5‐point analog scale was used, where scores 1 and 2 indicated low difficulty, 3 indicated moderate difficulty, and values 4 and 5 indicated high levels of difficulty [10]. The level of difficulty in diagnostic and treatment decision‐making using each imaging modality was recorded within each evaluator's clinical experience level group.

### 2.5 Statistical Methods

Statistical analyses were performed using Stata 13.0 software (Stata Corporation, LA, California, USA). McNemar's test was applied to compare the proportion of modified diagnostic options and treatment plans between the two assessments, while a binary logistic regression model was developed to investigate whether the evaluators' clinical level of experience influenced the likelihood of changes in diagnostic and therapeutic decisions between assessments. Additionally, the Wilcoxon signed‐rank test was employed to compare differences in self‐reported difficulty levels by evaluators when choosing diagnostic options and treatment plans based on CBCT and periapical radiography. Finally, an ordinal logistic regression model was used to examine the association between the type of imaging modality and the evaluators' clinical level of experience with the self‐reported difficulty level. All tests were conducted with a significance level of 5%.

## 3 Results

### 3.1 Diagnostic Assessment and Treatment Planning

**FIGURE 4:**
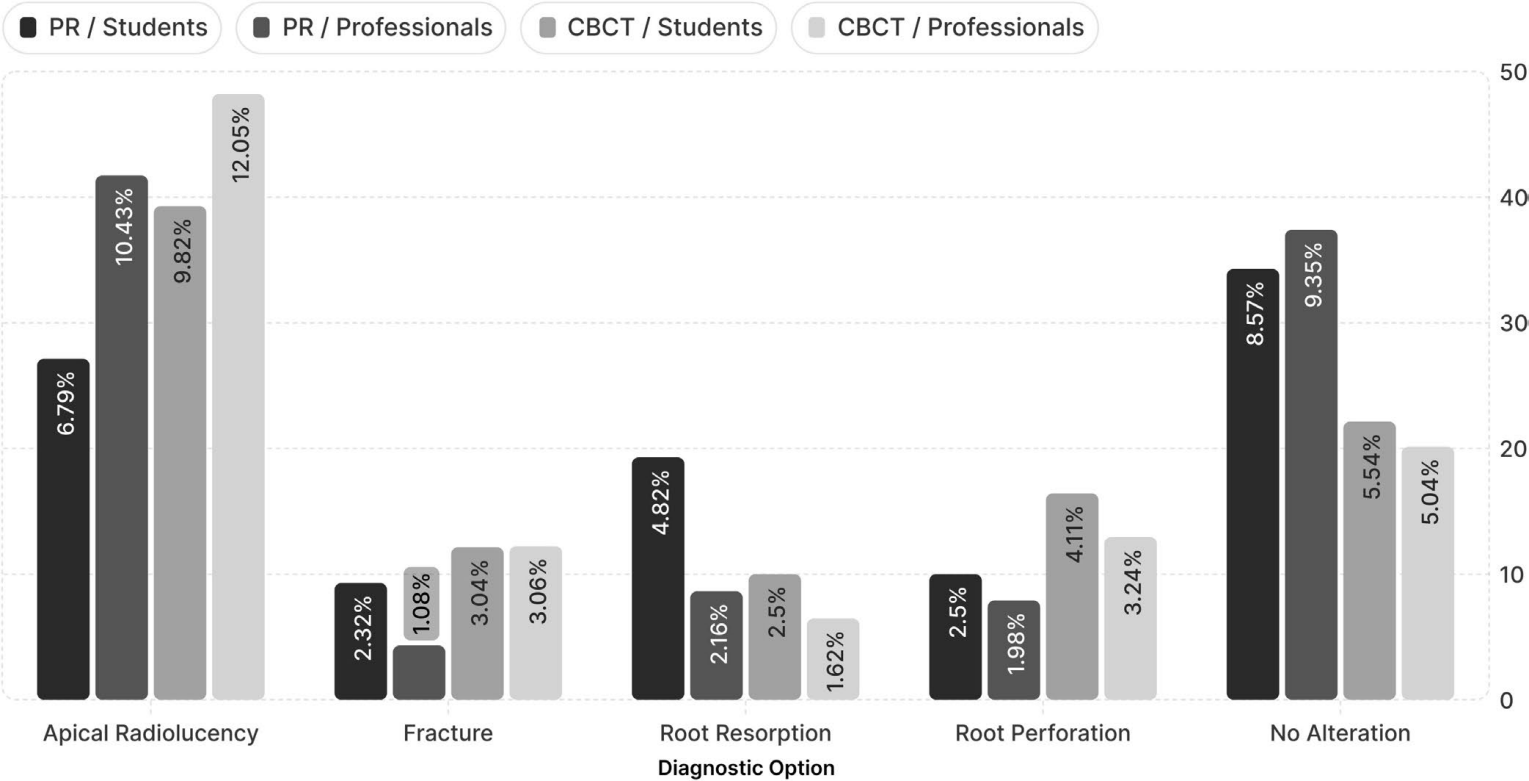
Distribution of diagnostic options between students and professionals in the first assessment, based on PR, and the second assessment, based on CBCT.

**FIGURE 5:**
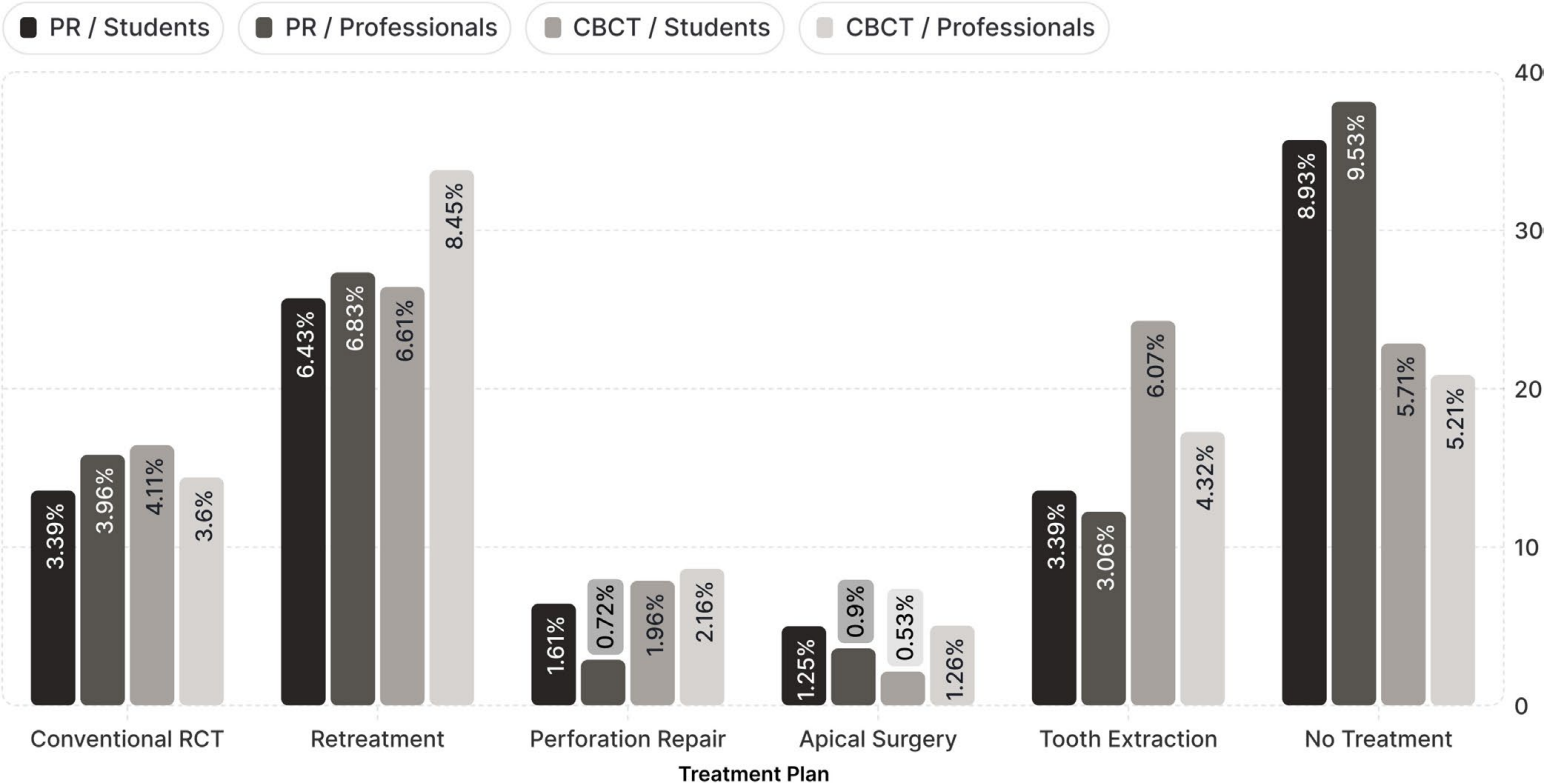
Distribution of treatment plan options between students and professionals in the first assessment, based on PR, and the second assessment, based on CBCT.

The proportions of diagnostic and treatment plan selections varied according to the imaging modality employed and the evaluator's level of experience (Figures 4 and 5).

Among undergraduate students, 32.9% of diagnostic decisions and 36.4% of treatment plans were altered during the second assessment compared to the first. In both outcomes, the proportions of change were statistically significant (*p* = 0.000). Moreover, the findings indicate that these evaluators were approximately 12 times more likely to modify their diagnostic or therapeutic decisions after reviewing CBCT images. Among professionals, the rate of change was even higher, with 45.7% of diagnostic decisions and 47.1% of treatment plans revised, and an odds ratio of approximately 17 (*p* = 0.000), reflecting a markedly increased likelihood of revising clinical decisions in light of the additional information provided by CBCT (Table 1).

**TABLE 1:** Changes in diagnosis and treatment planning after cone beam computed tomography (CBCT) analysis, according to evaluator's level of experience.

	Evaluator's level of experience	Cases changed *n* (%)^a^	*p* **	Proportion difference (95% CI)	OR
Diagnostic options	Students	46 (32.9%)	*p* = 0.000	0.329 (0.244–0.414)	11.98
	Professionals	64 (45.7%)	*p* = 0.000	0.457 (0.367–0.547)	16.85
Treatment plan	Students	51 (36.4%)	*p* = 0.000	0.364 (0.277–0.451)	13.33
	Professionals	66 (47.1%)	*p* = 0.000	0.471 (0.382–0.561)	17.40

Abbreviation: OR, odds ratio.

^a^ The change in diagnostic decisions and treatment plans refers to the comparison between the evaluation based on periapical radiographs (evaluation 1) and that based on CBCT (evaluation 2) for the same cases.

** *p*‐value according to McNemar's test.

For diagnostic options, professionals had approximately 42% lower odds of modifying their diagnostic choices when assessing CBCT images compared to students. This finding was statistically significant (*p* = 0.028), suggesting that clinical experience plays a role in diagnostic decision‐making for endodontic cases. In contrast, for treatment plans, the association did not reach statistical significance (*p* = 0.070), indicating that CBCT may influence the therapeutic decision‐making of both students and professionals to a similar extent in the evaluation of endodontic cases (Table 2).

**TABLE 2:** Association between evaluator's level of experience and the likelihood of diagnosis and treatment planning change after cone beam computed tomography (CBCT) analysis.

	Independent variable^a^	Coefficient (β)	Odds ratio (95% CI)	*p* **
Diagnostic options	Professionals	−0.543	0.58 (0.36–0.94)	0.028
Treatment plan	Professionals	−0.442	0.64 (0.40–1.04)	0.070

*Note:* Dependent variables: diagnostic options change (0 = no, 1 = yes); treatment plan changes (0 = no, 1 = yes).

^a^ Students were used as the reference category.

** *p* and Odds Ratio according to Logistic Regression Model.

### 3.2 Self‐Reported Difficulty

For both self‐reported difficulty in diagnostic decision‐making and treatment planning outcomes, there was a statistically significant increase in perceived difficulty among undergraduate students when using CBCT. In contrast, professionals reported a significant reduction in perceived difficulty with the use of CBCT (Table 3).

**TABLE 3:** Self‐reported difficulty in diagnosis options and treatment planning by students and professionals using periapical radiography (PR) and cone beam computed tomography (CBCT) for evaluator's level of experience.

Variable	Students		*p* *	Professionals		*p* *
	PR	CBCT		PR	CBCT	
Diagnostic options			0.000			0.019
Low (1–2)	35.25%	17.86%		35.04%	47.79%	
Moderate (3)	25.18%	20.71%		29.20%	31.62%	
High (4–5)	39.57%	61.43%		35.77%	20.59%	
Treatment plan			0.0011			0.0075
Low (1–2)	42.75%	31.43%		24.26%	39.71%	
Moderate 93)	18.84%	13.57%		42.65%	41.91%	
High (4–5)	38.41%	55.00%		33.09%	18.38%	

* *p*‐value according Wilcoxon signed‐rank test.

The perception of difficulty, in both diagnostic decision‐making and treatment planning, was significantly lower among professionals (*p* = 0.000), who exhibited a 56% and 47% reduction in the likelihood of reporting high levels of difficulty for diagnostic and treatment decisions, respectively, compared to undergraduate students, regardless of the imaging modality employed. These findings indicate that the evaluators' clinical experience was a more substantial determinant of the perceived complexity of endodontics than the imaging technology utilised in the evaluation (Table 4).

**TABLE 4:** Comparative analysis of Odds Ratio (OR) and significance levels for self‐reported difficulty in diagnosis and treatment planning.

Self‐reported difficulty	Independent variable	Coefficient (β)	Odds ratio (95% CI)	*p* ***
Diagnostic options	Exam^a^	0.141	1.15 (0.84–1.57)	0.374
	Evaluator^b^	−0.825	0.44 (0.32–0.60)	0.000
Treatment plan	Exam^a^	−0.048	0.95 (0.70–1.30)	0.764
	Evaluator^b^	−0.627	0.53 (0.39–0.73)	0.000

*Note:* Dependent variables: self‐report difficulty (0 = low, 1 = moderate, 2 = high) for diagnosis and therapeutic options.

^a^ Exam (periapical × CBCT).

^b^ Evaluator (students × professionals).

*** *p*‐value and Odds Ratio according to Ordinal Logistic Regression Model.

## 4 Discussion

This before‐after study assessed the influence of imaging modality and clinical experience on diagnostic options and treatment decision‐making in endodontics. While similar studies have been conducted, further investigation was necessary to better understand the discrepancies between students and experienced professionals. The findings reinforce the crucial role of imaging in endodontic evaluation and emphasise how variations in clinical training can impact decision‐making. Understanding these variations is essential for optimising diagnostic protocols and improving educational strategies, ensuring that CBCT is employed effectively and judiciously in clinical practice.

Regarding diagnosis, the use of CBCT led to statistically significant changes in decision‐making, altering diagnoses in 32.9% of cases among undergraduate students (*p* = 0.000) and in 45.7% of cases among professionals (*p* = 0.000). Previous studies have reported similar findings, with diagnostic changes ranging from 42% to 63.3% in evaluations conducted by experienced endodontists [2, 5]. Conversely, Al‐Salehia and Horner (2017), observed diagnostic changes in only a minority of cases when evaluating posterior teeth requiring root canal retreatment [13]. These variations in findings likely reflect methodological differences, particularly in the selection of cases and participants. The present study included a broader range of endodontic pathologies and complications and included evaluators with varying levels of clinical experience, allowing for a more comprehensive simulation of real‐world clinical scenarios.

CBCT has been shown to enhance the detection of periapical pathologies across several methodological approaches [5, 9, 17, 18]. Accordingly, in the present study, its use significantly improved the identification of periapical lesions, particularly among professionals. CBCT assessment reduced the selection of the “no alteration” diagnostic option while increasing the identification of “apical radiolucency”.

The use of CBCT significantly impacted treatment planning. After reviewing the tomographic images, professionals modified their plans in 47.1% of cases (*p* = 0.000) and undergraduate students in 36.4% (*p* = 0.000). Previous studies have reported varying modification rates following CBCT assessment, ranging from 24.3% to 62.2% [5, 7, 9, 10]. Despite this variability, a consistent finding across those studies is that CBCT provides additional diagnostic details that can lead to changes in clinical decision‐making. Our results are consistent with this pattern, suggesting that the added dimensional and structural information offered by CBCT contributes meaningfully to treatment planning in a substantial proportion of endodontic cases [5, 6].

Despite the various benefits of CBCT, its proper interpretation remains challenging for less experienced evaluators, a pattern reflected in multiple aspects of the present study [2, 9]. Students reported significantly greater difficulty when analysing cases with CBCT compared to PR, even though they altered their diagnoses and treatment plans less frequently than the professionals. Specifically, 61.43% of students reported high difficulty when establishing a diagnosis with CBCT (*p* = 0.000), and 55.00% did so when formulating a treatment plan (*p* = 0.0011). This apparent paradox suggests that while CBCT provides more detailed information, students may lack the diagnostic confidence or interpretive skills to translate this data into clinical decisions. In contrast, specialists were more likely to modify their decisions based on the tomographic information, potentially reflecting a greater readiness to integrate additional findings into their clinical reasoning. These findings align with previous research, such as Parker et al. (2017), which demonstrated that diagnostic accuracy in CBCT interpretation is strongly associated with the clinician's level of experience [19]. Additionally, students demonstrated a greater tendency to indicate tooth extraction after CBCT analysis compared to professionals. This may reflect a combination of factors: the enhanced visualisation provided by CBCT, particularly of root fractures, resorptions, and perforations, can prompt more invasive decisions, as suggested by previous studies [9, 10, 14]. However, in our sample, this tendency was observed predominantly among less experienced evaluators. It is possible that, when faced with complex tomographic findings and limited clinical experience, students interpret uncertain or ambiguous features as definitive indicators for extraction.

These findings reinforce the need for targeted education in CBCT interpretation and clinical decision‐making under diagnostic uncertainty, particularly among less experienced clinicians. Recent observational studies have examined the global adoption of emerging technologies in endodontics, reporting rates as high as 91.2% for usage of CBCT, yet, continuing professional education remains the primary training method for interpretation (69.2%) [20, 21]. As the integration of advanced imaging becomes increasingly routine, structured training in three‐dimensional imaging is essential for dental students. To address this need, the European Society of Endodontology advocates for integrating CBCT interpretation into undergraduate and postgraduate curricula ensuring that future clinicians are not only familiar with the technology but also prepared to interpret it critically and apply it judiciously in clinical decision‐making [8, 22].

While CBCT provides significant diagnostic advantages and may serve as a preoperative imaging tool in select cases, its use must be carefully weighed against its higher radiation exposure compared to PR [6]. The ESE, AAE and the AAOMR recommend that CBCT should only be considered after clinical evaluation and periapical radiographic assessment, ensuring that its benefits outweigh potential risks [3, 8]. When CBCT is indicated, optimising the field of view (FOV) is essential, as a limited FOV reduces both patient radiation exposure and the amount of data requiring interpretation [3, 18]. Qu et al. (2010) reported that a restricted FOV delivers a radiation dose comparable to three or four PR, making CBCT a viable alternative when multiple periapical images would otherwise be necessary [23]. However, proper training in CBCT interpretation is crucial to avoid misinterpretation or unnecessary imaging that could complicate clinical decision‐making.

Overall, evaluators reported difficulties in diagnosing cases and determining treatment plans, which may be attributed to the absence of clinical data during assessment, a recognised limitation of this study. However, this methodological choice allowed for a controlled comparison of imaging modalities and evaluator experience, aligning with that of Ee, Fayad, and Johnson (2014), who similarly isolated image interpretation from clinical findings to minimise bias [5]. Despite this study design, it is essential to reinforce that imaging should always serve as a complementary tool to clinical examination, rather than a standalone diagnostic method. Additionally, participants evaluated pre‐selected CBCT slices rather than navigating the full volumes. This approach was adopted to ensure that all evaluators interpreted the same diagnostic features, reducing variability due to differences in navigation skills or interpretation strategies, particularly relevant when comparing groups with distinct levels of clinical experience. Similar methodologies have been employed in recent endodontic studies [15, 16]. However, this resource may facilitate the interpretation by the professionals. To clarify this issue, future studies are recommended to assess the impact of providing either selected images or the full tomographic volume for unrestricted navigation on the perceived difficulty of CBCT interpretation by endodontic specialists.

According to the hierarchical model of imaging efficacy proposed by Fryback and Thornbury (1991), this study primarily contributes to diagnostic thinking efficacy (Level 3) and therapeutic efficacy (Level 4) [1]. Changes in diagnosis, perceived difficulty, and treatment planning between imaging modalities were used to assess how CBCT affects clinical reasoning and proposed interventions. Although the study did not evaluate patient outcomes or societal efficacy (Levels 5 and 6), these higher levels are built upon the foundational effects addressed in earlier stages, such as the present investigation. While caution is warranted when extrapolating these findings to full clinical contexts due to the absence of supporting clinical data, the study provides relevant insight into how CBCT may influence decision‐making processes under controlled conditions, particularly across different levels of clinical experience, and highlights potential implications for endodontic education and training in advanced imaging interpretation.

## 5 Conclusion

The findings of the present study highlight that the use of CBCT significantly altered diagnostic and therapeutic decisions in endodontic cases among both final‐year dental students and endodontic specialists. Notably, the evaluator's level of training played a key role in the decision‐making process: less experienced participants reported greater difficulty interpreting tomographic images, despite the additional information provided compared to PR. These results underscore the importance of emphasising training strategies for CBCT interpretation, particularly at the undergraduate level, given the growing integration of this imaging modality into endodontic practice.

## Author Contributions

**Tamyres Veleda Fonseca:** conceptualization; data curation; methodology; validation; roles/writing – original draft; and writing – review and editing. (ICMJE'S criteria: I; II; III; IV). **Gabriel Lima Braz:** formal analysis, methodology, validation; supervision; roles/writing – original draft; and writing – review and editing. (ICMJE'S criteria: I; II; III; IV). **Henrique Timm Vieira:** data curation; methodology; validation; supervision; roles/writing – original draft; and writing – review and editing. (ICMJE'S criteria: I; II; III; IV). **Nadia de Souza Ferreira:** conceptualisation; data curation; formal analysis; methodology; validation; supervision; roles/writing – original draft; and writing – review and editing. (ICMJE'S criteria: I; II; III; IV). **Melissa Feres Damian:** conceptualisation; data curation; formal analysis; methodology; validation; supervision; roles/writing – original draft; and writing – review and editing. (ICMJE'S criteria: I; II; III; IV).

## Disclosure

The authors have nothing to report.

## Ethics Statement

The research was approved by an institutional Research Ethics Committee of the Federal University of Pelotas (UFPel) (process no. 11567319.1.000.5318).

## Supporting information


**Table S1.** Reasons for referral for cone beam computed tomography.

## Data Availability

The data that support the findings of this study are available from the corresponding author upon reasonable request.
