# Supplementary material for: Do CBCT and Clinical Experience Impact Decision‐Making in Endodontic Diagnosis and Treatment Planning? A Before‐After Study
Source: Aust Endod J. 2025 Jun 19;51(2):474–82. doi: 10.1111/aej.12966 (PMC12351089; doi:10.1111/aej.12966)
Supplement: Supplementary file 1 — Table S1. Reasons for referral for cone beam computed tomography. [file AEJ-51-474-s001.docx]

| **Supplementary Table S1: Reasons for referral for Cone Beam Computed Tomography** | | |
| --- | --- | --- |
| **Clinical Diagnosis or reason of investigation** | **n** | **%** |
| Root fracture | 11 | 39.28 |
| Retreatment (Previous endodontic treatment) | 4 | 14.28 |
| Root resorption | 3 | 10.72 |
| Decision between retreatment or dental implant placement | 3 | 10.72 |
| Tooth crack | 2 | 7.15 |
| Retreatment (Previous endodontic treatment with metal or fiber post) | 1 | 3.57 |
| Calcified root canal | 1 | 3.57 |
| Investigation of Internal morphology | 1 | 3.57 |
| Measuring of apical lesion | 1 | 3.57 |
| Perforation | 1 | 3.57 |
| **Total** | 28 | 100 |
